# The Impact of Virtual Reality on Cardiopulmonary Function and Adherence in Cardiac Rehabilitation Patients: A Systematic Review and Meta-Analysis

**DOI:** 10.3390/healthcare13222969

**Published:** 2025-11-19

**Authors:** Qiqi Cheng, Feng Li, Qingyuan Zhang, Huidan Yu, Suqing Wang

**Affiliations:** 1 School of Nursing, Wuhan University, 115 Donghu Road, Wuchang District, Wuhan 430072, China; 2022283070023@whu.edu.cn; 2 Structural Heart Disease Center, ZhongNan Hospital of Wuhan University, 169 Donghu Road, Wuchang District, Wuhan 430071, China

**Keywords:** virtual reality, cardiac rehabilitation, cardiopulmonary function, quality of life, adherence, meta-analysis

## Abstract

**Purpose:** This systematic review aimed to evaluate the effectiveness of virtual reality (VR) technology in cardiac rehabilitation (CR) patients, focusing on cardiopulmonary function, quality of life, adherence, and satisfaction. **Methods:** Conducted following PRISMA guidelines and registered on PROSPERO (CRD42025643632), this study systematically searched PubMed, Web of Science, EMBASE, Cumulative Index to Nursing and Allied Health Literature (CINAHL), Cochrane Library, Scopus, CNKI, and Wanfang Data. Meta-analysis was performed using RevMan 5.4 to assess the impact of VR interventions on cardiopulmonary function, quality of life (QOL), adherence, and satisfaction compared to conventional CR programs. **Results:** Twenty studies were included. Meta-analysis revealed that VR improved the 6 min walk test distance (MD: 34.9, 95% CI: 24.43, 45.37; *p* < 0.00001, I^2^ = 67%) and QOL (SMD: 0.63, 95% CI: 0.09, 1.17; *p* = 0.002, I^2^ = 86%). However, evidence regarding adherence and satisfaction was inconclusive. **Conclusions:** VR technology outperforms traditional CR in enhancing cardiopulmonary function and quality of life. While it might improve patient adherence, further research is necessary to confirm these findings.

## 1. Introduction

Cardiovascular diseases (CVDs) represent the predominant cause of global mortality, exerting a profound economic strain on healthcare systems worldwide. According to the American Heart Association, CVDs accounted for approximately 19.41 million deaths annually, with healthcare expenditures in the United States alone reaching $417.9 billion in 2021 [1]. Beyond the financial burden, CVDs significantly impaired patients’ quality of life, necessitating effective and accessible interventions [2]. International guidelines include cardiac rehabilitation (CR) as a cornerstone of secondary prevention [3,4,5,6,7]. Integrating medical evaluation, supervised exercise, risk factor modification, and patient education, CR has been demonstrated to be cost-effective [8] and clinically beneficial, with a community observational study revealing a greater than 12% reduction in 10-year all-cause mortality among Coronary Artery Bypass Grafting (CABG) patients enrolled in CR programs [9]. Furthermore, outpatient cardiac rehabilitation has demonstrated a mortality and morbidity reduction of approximately 25% compared to standard care [10,11]. Despite these compelling benefits, CR utilization remained suboptimal, with referral and participation rates ranging from 20% to 30% among eligible patients [10], and only 34% of referred patients enrolling in programs [12]. Among nations with low to middle incomes, these challenges were exacerbated by resource limitations, insufficient CR infrastructure, and exclusion from national health insurance schemes [13].

Virtual reality (VR), an innovative and rapidly advancing field, offers a promising solution to these barriers. VR utilizes computer-generated, immersive, three-dimensional environments to provide sensory feedback, enhancing user engagement and motivation [2]. Recent studies have explored the application of VR in CR, demonstrating its potential to mitigate negative emotional conditions like anxiety, depression, and stress [14,15,16,17,18,19,20,21,22]. VR may advance health equity and be more cost-effective, given the limited resources and lack of inclusion in national health insurance schemes [2,23,24]. However, the impact of VR on cardiopulmonary function remains controversial. For instance, Chen et al. [14] proposed that virtual reality could enhance motor performance in patients engaging in CR, whereas Blasco-Peris et al. [25] reported contradictory findings. This divergence might be explained by the limited sample size in the latter investigation, potentially resulting in insufficient statistical power to identify a significant effect. And its effects on broader health indicators are yet to be conclusively established, such as quality of life, patient satisfaction, and adherence.

This study aimed to address these critical gaps by systematically evaluating the efficacy of VR-based CR programs in enhancing cardiorespiratory fitness, QOL, and adherence among cardiovascular patients, compared to traditional CR approaches. By synthesizing the latest evidence, this research sought to elucidate the potential of VR to redefine conventional paradigms in CR, offering insights that could redefine rehabilitation paradigms and improve patient outcomes. The findings hold profound implications for clinical practice, policy-making, and the broader adoption of digital health technologies in cardiovascular care, ultimately contributing to the global effort to reduce the burden of CVDs.

## 2. Methods

### 2.1. Study Design

This research was conducted in strict accordance with the Preferred Reporting Items for Systematic Reviews and Meta-Analyses (PRISMA) guidelines 2020 [26] and prospectively registered with PROSPERO (ID: CRD42025643632). A systematic methodology was implemented, including standardized search strategies, dual-independent screening, and quality assessment to ensure transparency and reproducibility.

### 2.2. Literature Search and Screening

Two researchers systematically and independently searched it in nine databases, including PubMed, Web of Science, Cumulative Index to Nursing and Allied Health Literature (CINAHL), Cochrane Library, Embase, Scopus, Wanfang Data, CNKI, and VIP database, from inception to 28 July 2025. The search method was in the form of mesh terms and keywords, such as “virtual reality” or “virtual gam*” or “virtual therapy*” or “Exergames*” or “Wii”; AND “Cardiovascular Disease” or “Cardiac Event*” or “Heart Disease” or “Cardiac Disorder*” or “Heart failure” or “cardiac rehabilitation” or “rehabilitation training” or “exercise training rehabilitation”, etc. In addition, we performed citation searches. The systematic retrieval process is provided in the Supplementary Table S1.

### 2.3. Eligibility Criteria

According to the PICOS principle, we set the inclusion and exclusion criteria. P (Population): Population with cardiovascular diseases involved in cardiac rehabilitation. I (Intervention): Intervention in the form of an exercise-based virtual reality rehabilitation program or exercise games, including immersive and non-immersive virtual environments. C (Comparison): Traditional cardiac rehabilitation or usual care. O (Outcomes): This study utilized the 6 min walk test (6MWT) and adherence as primary outcomes, with QOL and patient satisfaction assessed as secondary endpoints. The 6MWT was employed as a robust functional capacity indicator, reflecting cardiopulmonary fitness. This measure is widely adopted in clinical settings owing to its practicality, standardized protocol, and strong reproducibility [27,28]. Adherence was defined as the proportion of prescribed treatment sessions completed by the patient. Satisfaction was defined as the level of contentment with the treatment. S (Study design): A randomized controlled (RCT) design. Protocols, grey literature, and qualitative studies were excluded due to the exclusion criteria.

### 2.4. Data Extraction

First, all identified studies were imported into the reference management tool (NoteExpress V4.0). After removing repetitions, two researchers independently read the titles and abstracts one by one to obtain the preliminary results. Second, the full text should be reviewed against the eligibility criteria to select the relevant studies and extract data. Third, when a controversial article was encountered, a third researcher was involved in the discussion and consultation to make a decision.

### 2.5. Quality Evaluation

Initial assessments for risk of bias were conducted independently by two reviewers using the Cochrane Risk of Bias Tool 2 (RoB 2) [29]. The degree of agreement between reviewers was subsequently assessed, yielding a Cohen’s kappa statistic of 0.741 (*p* < 0.001), which indicates substantial consistency. To resolve disagreements, a third reviewer was consulted to reach a final consensus.

### 2.6. Data Integration and Analysis

Data analysis was performed using the software Review Manager (RevMan) version 5.4. For continuous outcomes, weighted mean difference (WMD) was used to assess 6MWT, and for the QOL that were assessed using different questionnaires, we used standardized mean difference (SMD) as the merging statistic and chi-square tests to determine inter-study heterogeneity. In accordance with Cochrane Handbook recommendations, heterogeneity was assessed through examination of study characteristics, sensitivity analyses, and subgroup analyses, with the sources of heterogeneity interpreted and reported. The confidence interval was 95%, and *p* < 0.05 indicated that the study was statistically significant.

## 3. Results

### 3.1. Study Selection

**Figure 1:**
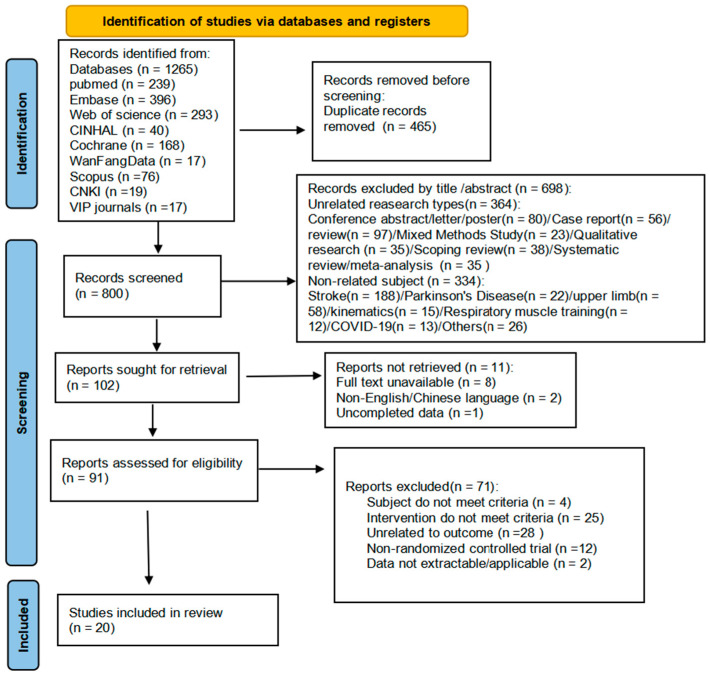
Flow diagram of study selection.

The database search obtained 1265 articles, of which 465 duplicates were removed, and 698 were excluded by reading the title and abstract. A total of 102 articles underwent preliminary screening based on their titles and abstracts; by reading the full text, we excluded 82 articles, 11 articles could not obtain the complete data, 28 were unrelated to outcome, 12 were non-randomized controlled trials, and 25 interventions did not meet criteria, 4 subjects did not meet criteria, 2 were data not extractable/applicable, and finally, 20 articles were included. The literature and the search flow chart are shown in Figure 1:

### 3.2. Study Characteristics

We included 20 studies from 2013 to 2025, including five studies [2,29,30,31,32] in the Americas (four in Brazil [2,29,30,31] and one in the United States [32]), seven in Europe [33,34,35,36,37,38,39] (two in Spain [35,38], two in Portugal [34,37], one in Ireland [33], one in Finland [39], one in Sweden [36]), and eight in Asia [40,41,42,43,44,45,46,47] (four in China [40,41,42,43,47], two in Thailand [45,46], and one in Japan [44]). The study population was mainly ischemic heart disease, heart failure, patients after PCI, or patients undergoing cardiac rehabilitation, etc. Six studies [2,30,31,34,35,37] used the Kinect console for intervention; two studies used [33,36] Nintendo Wii games; one study [32] used the Bionautica Trails system, a biotrajectory system developed in the United States; one study [45] used Toucher, an upper limb movement software developed in Singapore; one study [44] used a balance movement assist robot developed in Japan; and one study [47] used the PICO neo 3, a Chinese device; one [39] employed novel technology-equipped tablets in Finnish households; and one study [43] use BioMaster Virtual scenario interactive training system in China. Another four studies [29,41,42,46] did not describe the type of VR device used. The studies are characterized in Table 1. VR intervention measures and control group details are given in Table S2.

### 3.3. Risk Assessment of Quality Bias

**Figure 2:**
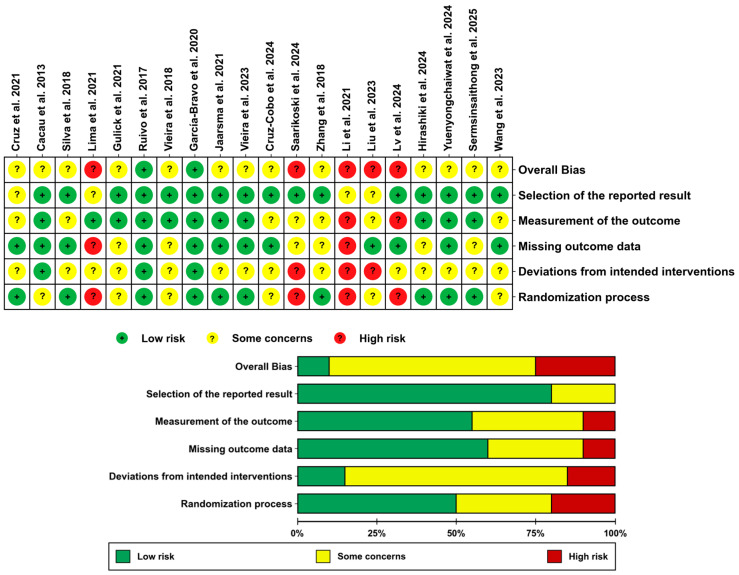
Bias risk summary [2,29,30,31,32,33,34,35,36,37,38,39,40,41,42,43,44,45,46,47].

Figure 2 presents all incorporated studies’ methodological quality evaluation results, detailing potential bias risks. Two studies [34,36] were at low risk of bias, thirteen studies [2,29,30,32,34,36,37,38,40,44,45,46,47] were at some concerns, and five [31,39,41,42,43] were assessed as high risk for bias. Of the included studies, 61.1% studies reported with low randomization bias; however, only 22.2% of the studies in domain 2, “Deviations from intended interventions”, reported low randomization bias as a result of VR being difficult to maintain blinding of the subjects and caregivers. In domain 3 “Missing outcome data” and domain 4 “Measurement of the outcome”, there are 63.9% studies that showed risk of bias and 30.6% studies reported some concerns. A total of 86.1% of studies showed a low risk of bias in domain 5 “Selection of the reported result”. The details are shown in Figure 2.

### 3.4. Results from the Analysis of Variables

#### 3.4.1. Cardiopulmonary Function

**Figure 3:**
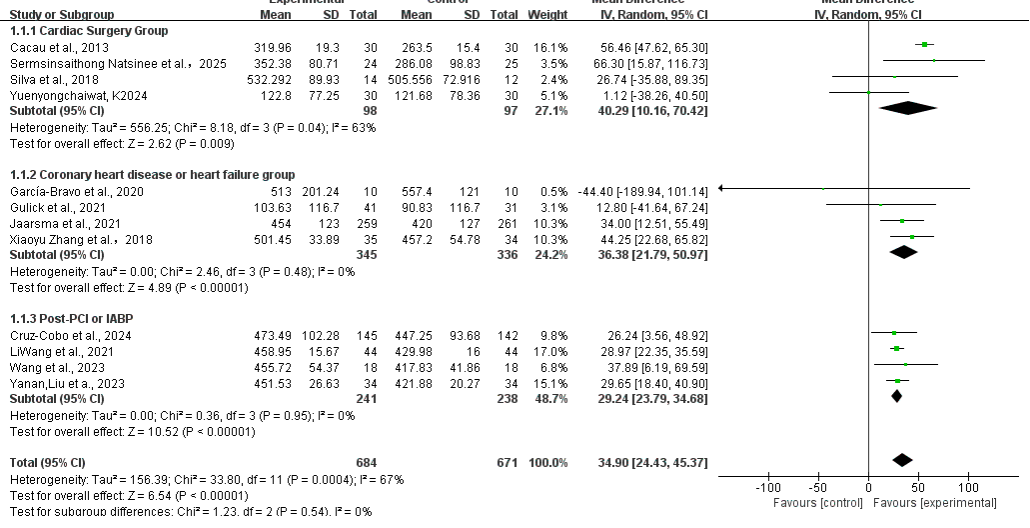
Meta-analysis of the 6 min walking distance in summary [29,30,32,35,36,38,40,41,42,45,46,47]. Green squares: study weight; horizontal lines: 95% confidence intervals; diamond: pooled effect.

Figure 3 presents the pooled analysis of 6 min walking distance (6MWD) from twelve randomized controlled trials (n = 1335) [29,30,32,35,36,38,40,41,42,45,46,47] (MD: 34.9, 95% CI: 24.43, 45.37; *p* = 0.0004), though with substantial heterogeneity (I^2^ = 67%). Subgroup analyses revealed significant improvements in Cardiac Surgery Group (MD: 40.29 m, 95% CI: 10.16 to 70.42; *p* = 0.009; I^2^ = 63%), Coronary heart disease or heart failure group (MD: 36.38 m, 95% CI: 21.79 to 50.97; *p* < 0.00001; I^2^ = 0%), and Post-PCI or IABP group (MD: 29.24 m, 95% CI: 23.79 to 34.68; *p* < 0.00001; I^2^ = 0%). Subgroup differences were substantial (I^2^ = 0%), suggesting condition-specific efficacy. We omitted each study one at a time to perform a sensitivity analysis. The results showed that I^2^ changed from 67% to 0% and *p <* 0.00001 in Cacau et al. 2013 [29], which might explain the source of heterogeneity. The results are shown in Figure S1.

#### 3.4.2. Quality of Life

**Figure 4:**
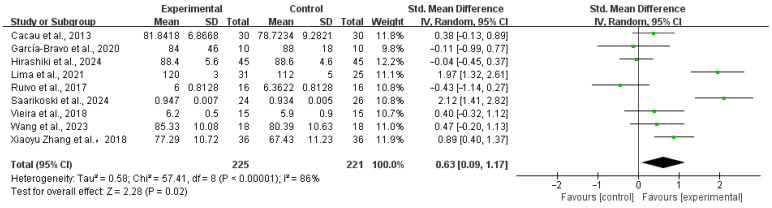
Meta-analysis of the QOL in summary [29,31,33,34,35,39,40,44,47]. Green squares: study weight; horizontal lines: 95% confidence intervals; diamond: pooled effect.

Nine studies [29,31,33,34,35,39,40,44,47,48] reported the effect on quality of life. One study [35] was excluded from the meta-analysis due to a standard deviation of zero, which violates its statistical assumptions. Three studies [29,31,44] used the Functional Independence Measure (FIM) scale for measurement, three studies [35,40,47] used the SF-36 questionnaire, two studies [33,34] used the MacNew questionnaire, and one study [39] used the 15-D questionnaire. The results indicated that virtual reality technology enhanced the QOL for CR patients. SMD: 0.63,95% CI:0.09, 1.17; *p* = 0.0002, I^2^ = 86%, as shown in Figure 4. Sensitivity analysis employing the leave-one-out method did not pinpoint the origin of heterogeneity. We intended to conduct subgroup analyses according to different measurement scales. However, due to an insufficient number of studies (fewer than three) in certain subgroups (e.g., the ‘Macnew’ and ‘15-D’ subgroups), quantitative meta-analysis was not conducted for these groups to ensure statistical robustness.

#### 3.4.3. Adherence

Seven studies [2,32,33,35,37,39,43] addressed adherence; the findings are suggestive of a potential benefit of virtual reality (VR) for improving adherence among CR patients, despite the lack of meta-analysis due to the different measurements. Adherence in four studies [2,33,37,39] was calculated as the percentage of patients attending the CR. One study [32] measured it by the number of people completing the recommended number of treatments by stage, and one study [43] categorized participants by adherence level (full, partial, non-adherent) and reported the proportion of each category as a percentage of the total population. Another study [35] did not report the results, although it described calculating by attendance at treatment. The impact on patient adherence outcomes is shown in Table 2.

#### 3.4.4. Satisfaction

Only two of the included studies [32,38] reported patient satisfaction scores, and the results of both studies indicated that patients responded more positively to treatment, with no statistical difference between in the virtual reality and control groups.

## 4. Discussion

This systematic review and meta-analysis aimed to evaluate the efficacy of virtual reality (VR)-based cardiac rehabilitation (CR) in improving cardiopulmonary function, QOL, adherence, and satisfaction among patients with cardiovascular diseases (CVDs). Our findings demonstrated that VR may improve cardiorespiratory fitness, particularly in patients who received early-phase intervention. However, the effect on quality of life requires cautious interpretation. And the evidence regarding adherence and satisfaction remains inconclusive. Compared with the previous systematic evaluation, the strength of this systematic review included adherence as the primary outcome measure and assessed the impact of the application of VR technology on adherence to cardiac rehabilitation. Although the progress of cardiopulmonary function and QOL was analyzed in the previous systematic evaluation, few studies were included, and the conclusions were inconsistent. Four studies [14,17,18,19] showed a possible improvement in cardiopulmonary function; three studies [20,25,49] showed no statistical difference and were unproven in terms of quality of life. Therefore, this study comprehensively summarized the evidence on cardiopulmonary function and QOL, increased the number of included studies, and improved the quality of the evidence. We also added analyses on adherence and satisfaction, which were not commonly found in previous systematic reviews.

### 4.1. Cardiopulmonary Function

The improvement in cardiorespiratory fitness, as measured by the 6 min walk test (6MWT), is a central finding of this study. The pooled analysis indicates that VR-based CR programs significantly outperform traditional CR in enhancing exercise capacity. These results are consistent with the findings of previous studies, which suggest that VR’s immersive and interactive nature can motivate patients to engage more actively in rehabilitation exercises [18]. Notably, subgroup analyses revealed that patients with cardiac surgery (MD: 40.29) and coronary heart disease or heart failure (MD: 36.38) benefited more than post-PCI (MD: 29.24). A potential factor contributing to these findings is the early initiation of the intervention [29,45]. This supports the earlier propositions put forth by several researchers [25,50]. The heterogeneity (I^2^ = 0%) and *p* = 0.46 in these subgroups strengthen the reliability of these findings. The physiological mechanisms by which VR interventions enhance patients’ cardiopulmonary function remain incompletely understood. Evidence from neurorehabilitation indicates that VR modulates neuroplasticity through sensory feedback, motor learning, and cognitive engagement [51]. Paralleling the neural activation mechanisms of portable neuromodulation techniques such as FES, tSCS, and tDCS [52]. Recent studies [53] further indicated that VR environments—even at constant exercise intensity—can elicit elevated subjective fatigue and cardiovascular responses through immersive scenarios (e.g., virtual slopes). Additionally, the visual stimuli provided by virtual reality can assist in regulating the brain’s cognitive systems, thereby influencing patients’ perceptions [54], and indirectly motivating them to increase their exercise engagement. In the future, more quantitative research can be added to further explore the physiological mechanisms by which VR affects cardiopulmonary function.

### 4.2. Quality of Life (QOL)

QOL is a crucial prognostic indicator of cardiovascular disease. Our study found that virtual reality intervention demonstrated a small and statistically marginal improvement in patient QOL, with considerable heterogeneity observed across the included studies. Nine studies contributed data to the meta-analysis on this outcome measure. Three studies [35,40,47] analyzed the impact on QOL on eight dimensions, including somatic functioning, role limitations, pain, socialization, and mental health, and reported higher scores in the intervention group. Evidence regarding between-group differences, especially in mental health outcomes, is limited to a single study [40], highlighting the need for further investigation to confirm these results. In contrast, three studies [35,40,47] assessed the total FIM scores, and no significant difference was observed between the two groups. In previous systematic reviews, Blasco-Peris et al. [25] and Peinado-Rubia et al. [17] conducted meta-analyses on QOL and showed small, non-statistically significant improvements in QOL, which is consistent with the findings in our analysis. Differently, our study added three studies that used FIM as a measurement tool, yielding statistically different results. Subgroup analyses were not conducted in this study due to limited data. Future research should incorporate standardized core outcome sets to further evaluate the effect of VR on QOL in CR patients.

### 4.3. Adherence

The role of VR in improving CR adherence remains inconclusive. This systematic review was not quantitatively analyzed due to limited studies and inconsistent measurement tools. Three studies [2,39,43] reported significant between-group differences, while Gulick et al. [32] observed higher completion rates in controls, attributing non-adherence primarily to external factors (e.g., work resumption, insurance issues). Two studies [33,37] were underpowered to detect a difference in median attendance, which might explain the nonsignificant finding. In addition, we retrieved two ongoing studies that may provide further insights [24,55], though preliminary evidence suggests a potential trend toward improved adherence with VR, consistent with findings in neurological rehabilitation [56]. Future research efforts should therefore aim to incorporate larger, diverse populations from multiple centers to corroborate these preliminary observations. Adherence is an important indicator used to measure the quality of cardiac rehabilitation, and there is no standardized measurement. Most studies have used adherence as measured by treatment attendance, while a few studies have reported dropout or withdrawal rates [57]. It demonstrated a dose–response correlation between participation in CR sessions and long-term results, with an estimated 1% reduction in mortality per CR session attended [58,59,60]. Evidence suggested a dose-dependent effect, where participation in thirty-six or more sessions correlated with significantly lower four-year risks of all-cause mortality and acute myocardial infarction relative to lower intervention frequencies. Thus, the American College of Cardiology/American Heart Association Task Force on Performance Measures recommends that ≥36 cardiac rehabilitation sessions be classified as a complete dose and used as the clinical target for cardiac rehabilitation quality measures [61]. In addition, the factors impacting patient adherence are complex. Studies have shown that higher social motivation, fewer sleep problems, and higher motor competence and self-efficacy are independently associated with patient motor adherence [62,63]. In terms of how virtual reality promotes adherence in rehabilitation, Micheluzzi et al. [64,65] proposed a mid-range theory to outline the mechanism of its effectiveness, which suggested that the effects of virtual displays on an individual consist of proximal and distal outcomes. Proximal outcomes were categorized as psychological (motivation, self-efficacy, mood) and physical (pain management, muscle strength) responses, and the distal outcomes were categorized as adherence, which interacted with each other. Virtual reality technology promotes the distraction of negative external stimuli associated with illness and healthcare environments, as well as the perception of positive emotions, through the activation of distal mediators, thus improving patient adherence. Interventional studies could be added to this in the future. The gamified elements of VR, such as real-time feedback and goal-oriented tasks, may enhance patient motivation and adherence to exercise regimens [66,67].

### 4.4. Satisfaction

Two studies [32,35] reflected patient satisfaction with the treatment, primarily attributed to the VR experience itself and its content design, though it was not comparable to traditional cardiac rehabilitation. Despite the high variability between studies, its positive impact on patient treatment has been supported by other literature. For example, Morgan H. et al. [65] utilized an immersive VR video for preoperative education of cardiac catheterization patients, which was effective in reducing preoperative anxiety and improving patient satisfaction. The application of VR as a supplementary approach for managing postoperative pain [68] could improve patient satisfaction, especially in patients undergoing laparoscopic surgery [69]. These findings emphasized VR’s potential in pain management, psychological care, and rehabilitation. However, given the subjective nature of satisfaction measures, future research should focus on comparative analyses across populations, VR program designs, and short-, medium-, and long-term outcomes.

Although the benefits of VR in enhancing cardiac rehabilitation outcomes are evident, practical implementation still faces certain challenges, such as limited acceptance among elderly patients and those with low awareness of VR technology [67]. Prolonged use of VR can cause motion sickness, along with high costs, limited reimbursement options, and insufficient training resources. Additionally, for patients with different conditions (such as atrial fibrillation), the anxiety stemming from the disease itself should be taken into account. Future research may draw upon successful rehabilitation models, such as the multidisciplinary approach proposed by Angelica Cersosimo et al. [70] integrating VR interventions into standard care pathways to deliver customized rehabilitation programs for patients across diverse settings, such as remote or home environments.

### 4.5. Limitations

This study has several limitations. The primary limitation of this review is the scarcity of available data across the included studies, which precluded quantitative meta-analysis and definitive conclusions, particularly regarding patient compliance and satisfaction, limiting the strength of our conclusions and reducing the overall evidence quality. Secondly, significant heterogeneity was observed across studies, particularly in cardiopulmonary function and QOL outcomes, likely due to variations in patient populations (e.g., participant demographics, baseline disease severity) and VR intervention protocols (including VR intervention parameters and types). Although Standardized Mean Differences (SMDs) were used to enable pooling, the combination of heterogeneous quality of life instruments may introduce bias. Thirdly, the lack of standardized measurement criteria for adherence and satisfaction posed analytical challenges. Although we attempted to minimize publication bias by extracting data from a consistent post-intervention period (2–3 months), some studies only reported short-term follow-up data. Additionally, to ensure the quality of evidence, this study did not include grey literature, which may have resulted in the omission of some unpublished data. Methodological enhancements in future studies should encompass sufficient statistical power through expanded recruitment, standardized data collection procedures, and proactive monitoring of VR-related side effects. Additionally, more rigorous studies are needed to elucidate VR’s impact on adherence and satisfaction while emphasizing the risk of bias assessment.

## 5. Conclusions

In conclusion, this study provides compelling evidence that VR-based CR can enhance cardiorespiratory fitness and QOL in CVD patients. However, the inconclusive findings on adherence and satisfaction highlight the need for further research to optimize VR interventions and address methodological challenges. By advancing our understanding of VR’s potential and limitations, this study contributes to the ongoing evolution of digital health technologies in cardiovascular care, offering a new idea for future research and clinical practice.

## Figures and Tables

**Table 1:** Study characteristics table.

Study (Year)	Country	Sample Size (E/C)	Age (Years) (E/C)	Participants	CR Phase	VR Type	Durration	Outcome
Cacau et al., 2013 [29]	Brazil	60 (30/30)	49.2 ± 2.6/52 ± 2.4	Post-cardiac surgery (CABG and/or HVR)	I	Not mentioned	3 days after surgery	①②
Ruivo et al., 2017 [33]	Ireland	32 (16/16)	59.4 ± 11.8/60.4 ± 8.5	CR patients	NA	NIVR	6 weeks	②③
Vieira et al., 2018 [34]	Portugal	22 (11/11)	55 ± 9.0/59 ± 5.8	Patients diagnosed with IHD undergoing CR	III	NIVR	24 weeks	②
Silva et al., 2018 [30]	Brazil	26 (14/12)	63.21 ± 8.27/63.75 ± 8.65	CVD (postoperative period of CABG, AMI, HTN, DM).	II	NIVR	8 weeks	①
Xiaoyu Zhang et al., 2018 [40]	China	72 (36/36)	64.6 ± 7.89/66.6 ± 7.52	Stable angina	I, II	NIVR	24 weeks	①②
García-Bravo et al., 2020 [35]	Spain	20 (10/10)	48.70 ± 6.66/53.7 ± 10.30	IHD	II	NIVR	8 weeks	①②④
Lima et al., 2021 [31]	Brazil	56 (31/25)	54 ± 8/51 ± 10	Post-CABG, via median sternotomy and cardiopulmonary bypass	I	NIVR	5 days	②
Cruz MMA et al., 2021 [2]	Brazil	61 (30/31)	63.27 ± 12.68/66.83 ± 10.93	CVD (HF, IHD, ASD, pericarditis, MFS) or with factors	II	NIVR	24 weeks	③
Gulick et al., 2021 [32]	United States	72 (41/31)	61 ± 9.9	CR patients	II	NIVR	8 weeks	①④
Jaarsma et al., 2021 [36]	Sweden	423 (207/216)	66 ± 12/67 ± 11	Heart failure	III	NIVR	48 weeks	①
Li, Wang et al., 2021 [41]	China	88 (44/44)	60.00 ± 1.50/60.10 ± 1.40	Post-PCI	III	Not mentioned	8 weeks	①
Wang et al., 2023 [47]	China	36 (18/18)	72.50 ± 6.16/72.61 ± 5.45	Elderly CHD, PCI	I	IVR	12 weeks	①②
Vieira et al., 2023 [37]	Portugal	46 (15/15/16)	55 ± 9.0/59 ± 5.8	CAD	III	NIVR	24 weeks	③
Yanan, Liu et al., 2023 [42]	China	68 (34/34)	51.28 ± 5.49/49.32 ± 6.53	Post-IABP	I, II	NIVR	2 weeks	①
Yuenyongchaiwat et al., 2024 [45]	Thailand	60 (30/30)	63.20 ± 9.57/64.43 ± 8.74	Post-OHS	I	NIVR	hospitalization	①
Hirashiki et al., 2024 [44]	Japan	90 (45/45)	78 ± 6/79 ± 6	Elderly CVD	II	NIVR	16 weeks	②
Cruz-Cobo et al., 2024 [38]	Spain	287 (145/142)	61.13 ± 8.69/63.93 ± 8.41	CAD, post-PCI	I, II, III	NIVR	24 weeks	①
Saarikoski et al., 2024 [39]	Finland	50 (24/26)	60 ± 8/65 ± 8	ACS, post-PCI	II, III	NIVR	24 weeks	①②③
Luyi, Lv et al., 2024 [43]	China	436 (236/200)	51.64 ± 8.37/50.93 ± 8.64	Post-PCI	NA	IVR	12 weeks	③
Sermsinsaithong et al., 2025 [46]	Thailand	49 (24/25)	62.75 ± 7.97/50.08 ± 13.97	Post-OHS	II	NIVR	8 weeks	①

Note: CABG: Coronary Artery Bypass Grafting; HVR: Heart Valve Replacement; IHD: Ischemic Heart Disease; ASD: Atrial Septal Defect; CR: Cardiac Rehabilitation; MFS: Marfan syndrome; CHD: Coronary Heart Disease; AMI: Acute Myocardial Infarction; HTN: Hypertension; DM: Diabetes Mellitus; HF: Heart Failure; PCI: Percutaneous Coronary Intervention; CAD: Coronary Artery Disease; IABP: Intraaortic Balloon Pump; OHS: Open-Heart Surgery; CVD: Cardiovascular Disease; ACS: Acute Coronary Syndrome; VR: Virtual Reality; NIVR: Non-immersive Virtual Reality; IVR: Immersive Virtual Reality. ①: Exercise capacity; ②: Quality of life; ③: Adherence; ④: Satisfaction.

**Table 2:** Summary of adherence findings.

Studies	Tools for Measuring Adherence	Results
Ruivo et al., 2017 [33]	Attendance rate was defined as the number of attended sessions per patient divided by the total number of sessions in the program and expressed as a percentage.	Despite the dropout trend, the median individual attendance rate showed no difference between groups.
García-Bravo 2020 [35]	Adherence was defined as the percentage of attendance to both treatment modalities.	The results were not described
Cruz MMA et al., 2021 [2]	Adherence was calculated as the proportion of completed on-site CR sessions out of the 36 scheduled over the preceding 12 weeks (3 sessions/week).	Among patients who were included in the analysis, the attendance rate in the intervention group was 82.8% over 12 weeks of treatment. While those in the control group were 73.51%. Adherence differed significantly between the study groups.
Gulick et al., 2021 [32]	Attendance was evaluated through both the recommended number of sessions and the completion of at least 18 sessions.	In at least 18 sessions, 22 in the intervention group and control group accounted for 58% and 81%, respectively; In the number of treatments achieved or exceeded, 16 (42%) in the intervention group and 19 (70%) in the control group. Control group had significantly higher completion rates (*p* = 0.02; 95% CI 0.04–0.53), as did the comparison of the rates for completing 18 sessions (*p* = 0.046; 95% CI 0.00–0.47).
Vieira, Ágata et al., 2023 [37]	The adherence rate was derived from the exercise diary using the formula: (Number of sessions completed/72) × 100%.	In EG1 group, the average compliance rate was 82%, 70%, 77% in the first three months, last three months, and six month period; in EG2 group, the average compliance rates were 90%, 75%, 83%, respectively. No statistically significant difference between the two groups.
Saarikoski et al., 2024 [39]	Training compliance was measured separately for each exercise modality (aerobic and resistance) as the ratio of attended sessions to the total number of sessions scheduled during the six-month trial.	The realized participation rate was significantly higher in the experimental group, particularly in resistance training. Compared with the control group and experimental group, no significant difference was found in the weekly frequency of aerobic exercise (3.4 ± 1.2 vs. 3.4 ± 0.8). However, strength training sessions differed significantly between groups (0.9 ± 0.3 vs. 1.2 ± 0.4 per week; *p* < 0.05).
Luyi, Lv et al., 2024 [43]	Adherence was quantified using the following formula: (Number of sessions completed/Number of sessions prescribed) × 100%. The definitions are as follows: ① Full compliance was defined as the patient’s active completion of the exercise program ② Partial compliance was defined as patients who need to be supervised to complete the exercise program ③ Non-compliance indicated that the patients is unwilling or unable to complete the exercise program on time	The compliance of VR group before and after intervention was 69.49% (164/236) and 87.71% (207/236), (*p* < 0.001), and that of the control group was 68.50% (137/200) and 79.50% (159/200), (*p* < 0.001), respectively. The intervention group demonstrated a statistically superior outcome relative to the control group (*p* < 0.05).

## Data Availability

No new data were created or analyzed in this study. Data sharing is not applicable to this article.

## Supplementary Materials

The following supporting information can be downloaded at: https://www.mdpi.com/article/10.3390/healthcare13222969/s1, Table S1: The search strategy of the systematic review and meta-analysis; Table S2: Details of VR Intervention Measures and Control Group; Figure S1: Sensitivity analysis of the 6MWT in summary [29,30,32,35,36,38,40,41,42,45,46,47]. Green squares: study weight; horizontal lines: 95% confidence intervals; diamond: pooled effect.
