# Supplementary material for: The Impact of Virtual Reality on Cardiopulmonary Function and Adherence in Cardiac Rehabilitation Patients: A Systematic Review and Meta-Analysis
Source: Healthcare (Basel). 2025 Nov 19;13(22):2969. doi: 10.3390/healthcare13222969 (PMC12652452; doi:10.3390/healthcare13222969)
Supplement: Supplementary file 1 [file healthcare-13-02969-s001.zip › healthcare-3919577-supplementary.pdf]

Table S1: The search strategy of the systematic review and meta-analysis

Term	Mesh term	Key words
Virtual Reality	Virtual Reality<br>Virtual Reality Exposure Therapy<br>Exergaming<br>Augmented Reality	virtual reality<br>virtual real*<br>virtual-real*<br>virtual environment<br>virtual rehabilitation*<br>virtual gam*<br>virtual treatment<br>virtual system<br>virtual program<br>virtual object<br>virtual world*<br>virtual therap*<br>Exergames<br>Exergaming<br>Wii<br>Active video game<br>Video games
cardiac rehabilitation	cardiac rehabilitation	cardiac rehabilitation<br>heart rehabilitation<br>cardiovascular rehabilitation<br>rehabilitation training<br>exercise training rehabilitation<br>home-based cardiac rehabilitation

Term	Mesh term	Key words
虚拟现实 (Virtual Reality)	虚拟现实 (Virtual Reality)<br>虚拟现实暴露疗法(Virtual reality exposure therapy)	VR
心脏康复 (cardiac rehabilitation)	心脏康复 (cardiac rehabilitation)	心血管康复 (cardiovascular rehabilitation)

Search strategy

PubMed

#1 Search: (((Virtual Reality[MeSH Terms]) OR (Virtual Reality Exposure Therapy[MeSH Terms])) OR (Exergaming[MeSH Terms])) OR (Augmented Reality[MeSH Terms]) Sort by: Publication Date 11481

#2 Search: virtual reality[Title/Abstract] OR virtual real*[Title/Abstract] OR virtual-real*[Title/Abstract] OR virtual environment[Title/Abstract] OR virtual rehabilitation*[Title/Abstract] OR virtual gam*[Title/Abstract] OR virtual treatment[Title/Abstract] OR virtual system[Title/Abstract] OR virtual program[Title/Abstract] OR virtual object[Title/Abstract] OR virtual world*[Title/Abstract] OR virtual therap*[Title/Abstract] OR Exergames[Title/Abstract] OR Exergaming[Title/Abstract] OR Wii[Title/Abstract] OR Active video game[Title/Abstract] OR Video games[Title/Abstract] Sort by: Publication Date 33001

#1 OR #2

#3 Search: ((((Virtual Reality[MeSH Terms]) OR (Virtual Reality Exposure Therapy[MeSH Terms])) OR (Exergaming[MeSH Terms])) OR (Augmented Reality[MeSH Terms])) OR (virtual reality[Title/Abstract] OR virtual real*[Title/Abstract] OR virtual-real*[Title/Abstract] OR virtual environment[Title/Abstract] OR virtual rehabilitation*[Title/Abstract] OR virtual gam*[Title/Abstract] OR virtual treatment[Title/Abstract] OR virtual system[Title/Abstract] OR virtual program[Title/Abstract] OR virtual object[Title/Abstract] OR virtual world*[Title/Abstract] OR virtual therap*[Title/Abstract] OR Exergames[Title/Abstract] OR Exergaming[Title/Abstract] OR Wii[Title/Abstract] OR Active video game[Title/Abstract] OR Video games[Title/Abstract]) Sort by: Publication Date 35873

#4 Search: cardiac rehabilitation[MeSH Terms] Sort by: Publication Date 4464

#5 Search: cardiac rehabilitation[Title/Abstract] OR heart rehabilitation[Title/Abstract] OR cardiovascular rehabilitation[Title/Abstract] OR rehabilitation training[Title/Abstract] OR exercise training rehabilitation[Title/Abstract] OR home-based cardiac rehabilitation[Title/Abstract] Sort by: Publication Date 12981

#4 OR #5

#6 Search: (cardiac rehabilitation[MeSH Terms]) OR (cardiac rehabilitation[Title/Abstract] OR heart rehabilitation[Title/Abstract] OR cardiovascular rehabilitation[Title/Abstract] OR rehabilitation training[Title/Abstract] OR exercise training rehabilitation[Title/Abstract] OR home-based cardiac rehabilitation[Title/Abstract]) Sort by: Publication Date 13995

#3 AND #6

#7 Search: ((cardiac rehabilitation[MeSH Terms]) OR (cardiac rehabilitation[Title/Abstract] OR heart rehabilitation[Title/Abstract] OR cardiovascular rehabilitation[Title/Abstract] OR rehabilitation training[Title/Abstract] OR exercise training rehabilitation[Title/Abstract] OR home-based cardiac rehabilitation[Title/Abstract])) AND (((((Virtual Reality[MeSH Terms]) OR (Virtual Reality Exposure Therapy[MeSH Terms])) OR (Exergaming[MeSH Terms])) OR (Augmented Reality[MeSH Terms])) OR (virtual reality[Title/Abstract] OR virtual real*[Title/Abstract] OR virtual-real*[Title/Abstract] OR virtual environment[Title/Abstract] OR virtual rehabilitation*[Title/Abstract] OR virtual gam*[Title/Abstract] OR virtual treatment[Title/Abstract] OR virtual system[Title/Abstract] OR virtual program[Title/Abstract] OR virtual object[Title/Abstract] OR virtual world*[Title/Abstract] OR virtual therap*[Title/Abstract] OR Exergames[Title/Abstract] OR Exergaming[Title/Abstract] OR Wii[Title/Abstract] OR Active video game[Title/Abstract] OR Video games[Title/Abstract])) Sort by: Publication Date 239

EMBASE

No.	Query Results	Results	Date
#16.	#11 AND #15	396	28 Jul 2025

#15. #12 OR #14 27,358 28 Jul 2025

#14. 'cardiac rehabilitation':ab,kw,ti OR 'heart rehabilitation':ab,kw,ti OR 'cardiovascular rehabilitation':ab,kw,ti OR 'rehabilitation training':ab,kw,ti OR 'exercise training rehabilitation':ab,kw,ti OR 'home-based cardiac rehabilitation':ab,kw,ti 22,060 28 Jul 2025

#12. 'heart rehabilitation'/exp 20,048 28 Jul 2025

#11. #8 OR #10 58,333 28 Jul 2025

#10. 'virtual reality':ab,kw,ti OR 'virtual real*':ab,kw,ti OR 'virtual-real*':ab,kw,ti OR 'virtual environment':ab,kw,ti OR 'virtual rehabilitation*':ab,kw,ti OR 'virtual gam*':ab,kw,ti OR 'virtual treatment':ab,kw,ti OR 'virtual system':ab,kw,ti OR 'virtual program':ab,kw,ti OR 'virtual object':ab,kw,ti OR 'virtual world*':ab,kw,ti OR 'virtual therap*':ab,kw,ti OR 'exergames':ab,kw,ti OR 'exergaming':ab,kw,ti OR 'wii':ab,kw,ti OR 'active video game':ab,kw,ti OR 'video games':ab,kw,ti 43,311 28 Jul 2025

#8. #4 OR #5 OR #6 OR #7 41,594 28 Jul 2025

#7. 'augmented reality'/exp 4,672 28 Jul 2025

#6. 'exergaming'/exp 824 28 Jul 2025

#5. 'virtual reality exposure therapy'/exp 1,299 28 Jul 2025

#4. 'virtual reality'/exp 36,916 28 Jul 2025

WOS:

#1 TS=(Virtual Reality OR Virtual Reality Exposure Therapy OR Exergaming OR Augmented Reality) 186525

#2 TS=(virtual real* OR virtual-real* OR virtual environment OR virtual rehabilitation* OR virtual gam* OR virtual treatment OR virtual system OR virtual programOR virtual object OR virtual world* OR virtual therap* OR Exergames OR Exergaming OR Wii OR Active video game OR Video games) 582697

#3 #2 OR #1 602212

#4 TS=(cardiac rehabilitation OR heart rehabilitation OR cardiovascular rehabilitation OR home-based cardiac rehabilitation) 69770

#5 #4 AND #3 1347

#6 TS=(randomized controlled trial)

#7 #5 AND #6 293

CINAHL

S1. SU (“Virtual Reality” OR “Virtual Reality Exposure Therapy” OR “Exergaming” OR “Augmented Reality”) OR TI ("virtual reality" OR "virtual real*" OR "virtual-real*" OR "virtual environment" OR "virtual rehabilitation*" OR "virtual gam*" OR "virtual treatment" OR "virtual system" OR "virtual program" OR "virtual object" OR "virtual world*" OR "virtual therap*" OR "Exergames" OR "Exergaming" OR "Wii" OR "Active video game" OR "Video games")

S2. SU “cardiac rehabilitation” OR TI (“cardiac rehabilitation” OR “heart rehabilitation” OR “cardiovascular rehabilitation” OR “rehabilitation training” OR “exercise training rehabilitation” OR “home-based cardiac rehabilitation”)

S3 (SU (“Virtual Reality” OR “Virtual Reality Exposure Therapy” OR “Exergaming” OR “Augmented Reality”) OR TI ("virtual reality" OR "virtual real*" OR "virtual-real*" OR "virtual environment" OR "virtual rehabilitation*" OR "virtual gam*" OR "virtual treatment" OR "virtual system" OR "virtual program" OR "virtual object" OR "virtual world*" OR "virtual therap*" OR "Exergames" OR "Exergaming" OR "Wii" OR "Active video game" OR "Video games")) AND (SU “cardiac rehabilitation” OR TI (“cardiac rehabilitation” OR “heart rehabilitation” OR “cardiovascular rehabilitation” OR “rehabilitation training” OR “exercise training rehabilitation” OR “home-based cardiac rehabilitation”)) 40

SCOPUS

(TITLE-ABS-KEY("Exergam*" OR "Virtual Reality") AND TITLE-ABS-KEY("cardiac rehabilitation")) 76

Cochrane library

ID Search

#1 Virtual Reality 9204

#2 (virtual reality or virtual real* or virtual-real* or virtual environment or virtual rehabilitation* or virtual gam* or virtual treatment or virtual system or virtual program or virtual object or virtual world* or virtual therap* or Exergames Exergaming or Wii or Active video game or Video games):ti,ab,kw 16988

#3 #1 or #2 17226

#4 cardiac rehabilitation 6231

#5 #3 and #4 168

WanFang Data

主题:(虚拟现实 OR 虚拟现实暴露疗法 OR 虚拟现实, 教育性 OR VR) and 主题:(心脏康复 OR 心血管康复) 17

Subject: (Virtual Reality OR Virtual reality exposure therapy OR Virtual reality, educational OR VR) AND Subject:(cardiac rehabilitation OR cardiovascular rehabilitation) 17

CNKI

（主题:虚拟现实 + 虚拟现实技术 + 虚拟现实系统 + ‘虚拟现实(vr)’ +虚拟现实技术应用 +‘虚拟现实(vr)技术’） OR （主题：虚拟现实暴露疗法）AND（主题:心脏康复 +心脏康复护理 + 心脏康复治疗 + 心脏康复效果 + 心脏康复依从性 + 心脏康复运动） 19

(Subject: Virtual Reality + Virtual reality technology + Virtual reality system + ‘Virtual Reality(vr)’ + Virtual reality technology applications + ‘Virtual reality (vr) technology’) OR (Subject: Virtual Reality Exposure Therapy) AND (Subject:Cardiac Rehabilitation + cardiovascular rehabilitation care + Cardiac rehabilitation + Cardiac rehabilitation outcomes + Cardiac rehabilitation compliance + Cardiac rehabilitation exercise) 19

VIP

(((任意字段=虚拟现实 OR 任意字段=虚拟现实暴露疗法) OR 任意字段=虚拟现实，教育性) AND (任意字段=心脏康复 OR 任意字段=心血管康复)) 17

((((All fields = Virtual Reality OR All fields = Virtual Reality Exposure Therapy) OR All fields = Virtual Reality, Educational) AND (All fields = Cardiac Rehabilitation OR All fields = Cardiovascular Rehabilitation)) 17

Table S2. Details of VR Intervention Measures and Control Group.

Study	Interventions	Control
Cacau et al., 2013 [29]	Physical therapy using virtual reality, twice a day	Conventional physical therapy
Ruivo et al., 2017 [34]	The AVG group engaged in twice-weekly one-hour sessions of Wii Sports boxing and canoeing. From week 4, participants were asked to play a canoeing game against a colleague	Traditional CR Program
Vieira et al., 2018 [35]	Use with Kinect<br>VR exercise on top of routine cardiac rehabilitation (3 times/week)	Usual care
Silva et al., 2018 [30]	Use with Kinect<br>Play with two games (YourShape™ and Dance Central 3™ games), twice a week	Treadmills for aerobic exercise (30 min) and free weights for resistance exercise
Xiaoyu Zhang et al., 2018 [41]	Use the Situation interactive rehabilitation system, including angle trekking, waterfall, falling rocks, house building, shopping in the supermarket, racing, and other challenging games, 20–40 min daily, five times weekly. BaDuanJin exercise with identical frequency and duration.	Traditional CR Program
García-Bravo et al., 2020 [36]	Use the Microsoft XBOX ONE console and Kinect sensor for VR-based aerobic exercise twice a week	Conventional physical training
Lima et al., 2021 [31]	Conventional physiotherapy and the XBOX 360 platform with the game Kinect Sports Ultimate Collection, Table Tennis mode. 20 min a day	Conventional physiotherapy, twice a day
Cruz MMA et al., 2021 [2]	Use the Kinect console to project the game onto a white wall. Virtual reality exercise training is carried out through “shaping” exercise games. Two weeks of usual CR, including 1 week of VR, 3 times per week	Traditional CR Program includes 36 sessions, 3 times/week, 50 min each time, 15 min of warm-up, 30 min of

		exercise training, and 5 min of relaxation
Gulick et al., 2021 [32]	Use a virtual treadmill with a virtual track. Completing 18 to 36 sessions according to the level of risk (recommended by CR staff)	Standard of care therapy
Jaarsma et al., 2021 [37]	Use the exergame computer (Nintendo Wii®) with baseball, bowling, boxing, golf, and tennis sports at home 30 min daily, five days per week	A protocol-based physical activity advice from an HF team member and motivational telephone follow-up at 2, 4, 8, and 12 weeks
LiWang et al., 2021 [42]	Home exercise rehabilitation supported by virtual reality technology	Usual care
Wang et al., 2023 [48]	Use the PICO neo 3 VR rehabilitation training system (PICO, China), including six kinds of games(Fruit Ninja, boxing, tennis, golf, running, and cycling)	Usual care
Vieira et al., 2023 [38]	Use the Kinect. An expert-certified physical therapist with 5 years' experience designed the 30 min exercise protocol, administered 3 days/week.	Conventional care
Yanan, Liu et al., 2023 [43]	The virtual reality rehabilitation system is unclear<br>The main training program is city driving 15 min a day<br>Single bridge movement and double bridge movement. Ten per group, 3 times a day	Usual care
Yuenyongchaiwat et al., 2024 [46]	Using Toucher Software ("Falling Snow", "Apple Tree" and "Hit the mole") for 30 min, once a day until hospital discharge	Conventional physical therapy, 30 min once a day until hospital discharge
Hirashiki et al., 2024 [45]	Balance exercise assist robot (BEAR) system, 1 session/week, 1 h/session, 4-month duration.	Usual cardiac rehabilitation
Cruz-Cobo et al., 2024 [39]	e-MOTIVATE app utilization: virtual classroom-based healthy lifestyle guidance (diet, physical activity, recommendations) aligned with treatment goals.	Usual care
Saarikoski et al., 2024 [40]	Adopted innovative tablet technologies were employed to precisely control and customize exercise duration, volume, and intensity.	Traditional trianing
Luyi, Lv et al., 2024 [44]	Watch health education videos using immersive VR glasses twice a day, 10–15 min each time	Usual health education
Sermsinsaithong et al., 2025 [47]	Interaction with virtual environment through 2D screen program 2–3 times a week	No rehabilitation program, but a workout manual were provided

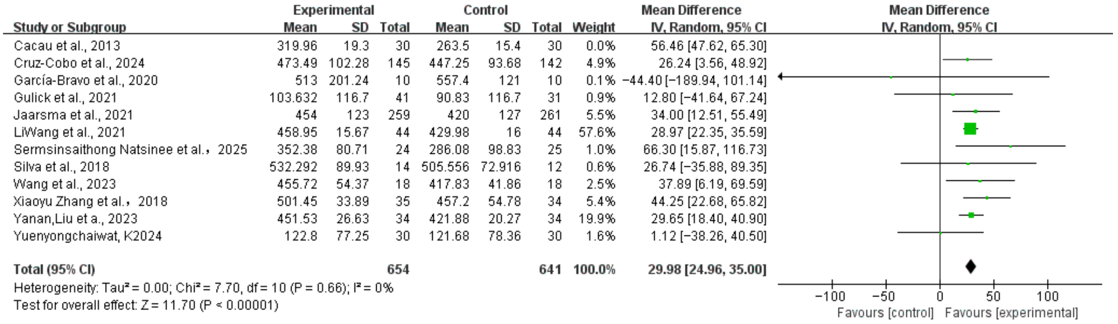

Figure S1. Sensitivity analysis of the 6MWT in summary [29,30,32,35,36,38,40–42,45–47]. Green squares: study weight; horizontal lines: 95% confidence intervals; diamond: pooled effect.
